# Notes and News

**Published:** 1893-12-02

**Authors:** 


### Notes and News.

Collected and Communicated.

The Council of the Harveian Society have departed somewhat from their usual routine by inviting an exponent of sanitary science to deliver the Harveian Lectures for the present year. The course will be undertaken by Professor Corfield, who takes for his subject, "Disease and Defective House Sanitation." The lectures will be delivered on December 7th, 14th, and 21st. The attendance of all members of the medical profession will be welcomed.

An amusing combat between two surgeons took place at a recent meeting of the French Academy of Medicine. Both were men of eminence, one being Professor Péan and the other Professor Verneuil. The subject of dispute was as to the method of performing a certain operation and as to the use of certain instruments. Professor Péan was for proving his ground by practical demonstration, and challenged his adversary to peform the operation in question publicly with the instruments customarily used by him. Professor Verneuil refused to treat the subject in the manner desired by Professor Péan as beneath his dignity.

Quite one of the funniest and most impracticable suggestions for the employment of the unemployed comes from the London Reform Union. This association actually made an application that the cleansing and painting of the hospitals should be relegated to the winter months in order to provide work for the class of workmen whom this season of the year is thrown out of occupation. It has been objected to by sentimentalists that the poor should be utilised as subjects for study, even to benefit the whole race, but the sacrifice asked of them by their own class is, undoubtedly, cruel, and the advantages to the few all out of proportion to the loss and risk it would entail on the many. It is needless to say that the application was rejected by those who have the true interests of the sick poor at heart.

The sale of the goods of defaulting vaccinators which has been taking place at Stroud created a great deal of excitement in the neighbourhood. A force of about forty constables was drafted into the town over and above those available in the locality. Eggs were thrown at the auctioneer, and windows of the Town Hall, where the sale was held, were broken. The crowd did not disperse until it was announced that the goods should not be removed until legal opinion on the sale had been given.

Gray's Inn Road can hardly be described as a picturesque thoroughfare, but its usefulness counterbalances its lack of beauty. The Royal Free Hospital one of its most noteworthy features, is a centre of interest to the surrounding juvenile population just now. Boys and brickbats have a natural affinity, and there is a great show of both at present, for the familiar old frontage has been demolished to make way for commodious offices and to give more space for the reception of patients. A better casualty room was sorely needed, and doubt ess adequate accommodation and shelter will soon fall to the lot of the newcomers, stricken by disease or sudden accidents. The needs of patients, staff, and students appear to be receiving due consideration, but what about the nurses? There is a small fund accumulating to furnish their new sitting-room, made up of little sums quietly and unostentatiously handed in for the purpose. The long, dreary dormitories at the Royal Free Hospital leave much to be desired, but they might be divided up into pleasant and distinct sleeping rooms if sufficient money were forthcoming to admit of adequate alterations being made.

If Sir James Crichton Browne and Dr. Neil Carmichael, of Glasgow, have by their strong utterances in relation to defective plumbing helped forward improvement in this direction, they must for ever be regarded in the light of public benefactors. The meeting at which the subject was brought forward was held at Dumfries, but we trust that through the medium of the Press their words will reach a larger field. All householders know the inestimable annoyance of bad plumbing, a class of work so generally inefficiently executed that it is almost a proverb that the visit of the plumber to repair one damage is usually followed by another to rectify the damage in some other direction caused by him in his first visit. A change of plumbers, too, almost invariably reveals shockingly bad work done by a previous plumber. Yet what protection does the public possess—what means have they of detecting bad workmanship? The discovered evils must be small in comparison to the undiscovered ones. Investigation is seldom complete until preceded by some calamity. Efficient plumbing is certainly a matter of national importance. Sanitation cannot be safely left in the hands of the individual, as however careful we ourselves may be, we always run the risk of less watchfulness on the part of our neighbours, many householders regarding a question of drainage as a personal reflection on themselves. If all master plumbers were held responsible for the work executed by their men, on pain of a fine, some sort of security might be felt by the public.

